# Address of B. Oscar Doyle, D. D. S., on Retiring from the Presidency of the Kentucky State Dental Association at Lexington, June, 3d. ’75

**Published:** 1875-10

**Authors:** 


### ADDRESS OF B. OSCAR DOYLE, D. D. S., ON RETIRING FROM THE PRESIDENCY OF THE KENTUCKY STATE DENTAL ASSOCIATION AT LEXINGTON, June, 3d, '75.

Gentlemen:—Custom has made it obligatory upon the presiding officers, in retiring from the executive chair, to deliver an address appropriate to such an occasion, but first of all before taking a retrospective glance of the year which has past, or considering matters which belong to our profession, it is incumbent upon us to acknowledge our thanks to Him whose eye is ever upon us, and without whose assistance, all human efforts are vain and profitless. Let us therefore give God the praise, for all the privileges and blessings which we enjoy, and ask his influence to be with us, to guide us in our deliberations, and when the annual meeting shall have closed to remain with us in our private walks.

The association and the members are one year older than when we last had the pleasure of meeting, and we may reasonably ask: Has this time wrought any material change with any of us? None will deny that the science of dentistry has advanced in this time, while there has not been very many new discoveries in any of the departments, yet many of the new theories have been tested, and *investigation*, seems to be the prevailing idea. So we may reasonably expect the future to be rich in valuable discoveries, and improved appliances, all of which will increase our store of knowledge, extend our usefulness, and mitigate much of the suffering connected with the practice of our specialty. The progressive members of the profession find an arduous task to keep pace, with the many practical and theoretical ideas advanced, and can only investigate, and hold fast to that which is good. Prominent amongst the experiments of the past year is the attempt to produce a vulcanizable gum, from one of the products of the common milk weed, this experiment while not yet perfected is in such a state of forwardness as to promise a perfect substitute for Rubber, without any of its objectionable features, and at the same time a perfect relief from the onerous exactions of the Rubber Co. Monopoly.

The experiments which have been conducted to ascertain the reliability of amalgams as a material for filling teeth, have resulted disastrously to nearly all of the different amalgams, subjected to the tests, and proves beyond cavil, that outside of the deleterious effects, supposed to result from the metallic mercury in the filling, that this material is almost totally unreliable, as an agent for the arrest of decay, on account of the shrinkage which takes place during consolidation, and in no case can permanent results be expected, unless the greatest care is exercised during its introduction into the cavity, and the material thoroughly condensed, and burnished, after consolidation has taken place. It seems about time that this material should be placed on the "retired list" it having so signally failed to prove itself valuable, except when manipulated with almost as much skill, and the expenditure of fully one half as much time as is necessary for the introduction of gold.

Tin foil has stood not only experiment, but the test of time, and answers well in all cavities not exposed to attrition.

But all of these and other experiments and discoveries which have come to notice during the year, are no doubt familiar to most of you, but before leaving the subject of improvements, I desire to bring the subject of Barnum's Rubber Dam, to the notice of those who have never learned its use. The many improvements in appliances to facilitate its use, many of which have been perfected recently, make its use comparatively easy, it needs no recommendation when it has once been used, operations, that without it are below the medium, or cannot be performed at all, are with its aid performed with a certainty of success to be obtained by no other means. My advice is "try it."

So far as the membership of this association is concerned, peace and quiet has reigned since our last annual meeting. It is my desire officially, to call attention to the deplorable condition of the large portion of the profession, in this state, it is well and personally known to every member, that empiricism," stalks abroad through the length and breadth of this commonwealth, seeking victims, and that the grossest mal-practice is perpetrated in the name of dentistry by men calling themselves "doctors," and who are entirely destitute of either medical or dental education, there is surely some remedy by which this evil can be lessened. The medical profession have a state law, which requires practitioners who are not graduates, to undergo an examination before a board of examiners appointed for that purpose, and this has caused many a self styled "doctor" to seek pastures new. In our profession, every man who chooses to style himself "dentist" immediatelly has the tittle of "doctor" bestowed, either by himself or a generous public, and the regular profession makes no distinction, between those who receive degrees from competent authority, and those who appropriate it unlawfully, all are spoken of as "doctor." In the present day of advancement and opportunity for education, there is no excuse whatever for any young man entering the practice of our specialty, without a thorough and practical education, to be obtained in any of our dental schools, and it is certainly the duty of the regular profession to discountenance; all such attempts as well as the unlawful appropriation of titles. But the great trouble has always been, that the members of the profession, who have endeavored to fully prepare themselves for honorable practice are content to settle down, happy in the confidence of their patrons, and with time so fully occupied, by that portion of the public, who appreciate *skill* and *integrity*, that they have no time to attend to such matters as these, and some even believe that the "empiric" serves a valuable purpose by way of comparison.

One year ago, at your hands, I received this, the highest in your power to bestow. In my official intercourse with the members I have endeavored to fulfill, to the best of my ability, the duties devolving upon me, friends and others alike; have been treated with that courtesy due from an official, and in surrendering the ch[illegible]r to my worthy successor, I have the proud satisfaction of knowing that it is surrendered free of tarnish. And now my brothers of the association, I will close by again thanking you for the confidence and honor confided to my keeping, and hope that each and every one of you, may be eminently successful in your efforts in this our chosen profession, that we may have the pleasure of meeting and mingling our thoughts together for many many years, and discord may never be heard in our councils, but that all our works may redound to the glory of God, the good of our fellow men, and the elevation of the dental profession.

